# Mesh Migration into the J-Pouch in a Patient with Post-Ulcerative Colitis Colectomy: A Case Report and Literature Review

**DOI:** 10.1155/2017/3617476

**Published:** 2017-11-23

**Authors:** Asem Ghanim, Benjamin Smood, Joseph Martinez, Melanie S. Morris, John R. Porterfield

**Affiliations:** ^1^Department of Surgery, University of Alabama at Birmingham, Birmingham, AL, USA; ^2^School of Medicine, University of Alabama at Birmingham, Birmingham, AL, USA

## Abstract

Mesh repair offers advantages like lower postsurgical pain and earlier return to work. Thus, it has become a widely used treatment option. Here, we present the first case report of a mesh migration into a J-pouch in a patient with history of ulcerative colitis who underwent total abdominal colectomy with J-pouch and ileoanal anastomosis and a subsequent laparoscopic ventral hernia repair with mesh.

## 1. Introduction

Incisional ventral hernia remains a common complication of abdominal surgery [1, 2]; its incidence is estimated at 10–15% with a recurrence of 20–45% [1, 2]. As such, patients undergoing J-pouch construction are at risk for hernia development and complications of the subsequent repair.

Complications often include infection, seroma, recurrence, or rejection but can include fistulas, erosions, and rarely mesh migration [3–5]. However, the low incidence of serious complications is likely underestimated due to the lack of long-term studies [6]. Several cases have reported bowel obstruction as a result of mesh migration [7, 8]. Although mesh migration to the colon has been reported [9, 10], to our knowledge, this is the first documented patient presentation with mesh migration into a J-pouch following incisional ventral hernia repair.

## 2. Case Report

A 43-year-old Hispanic male patient presented to the emergency department with intermittent lower abdominal pain and diarrhea. The patient had a previous history of ulcerative colitis and total abdominal colectomy with J-pouch and ileoanal anastomosis (2012) and a laparoscopic incisional ventral hernia repair with mesh (2015) in another institution.

**Figure 1:**
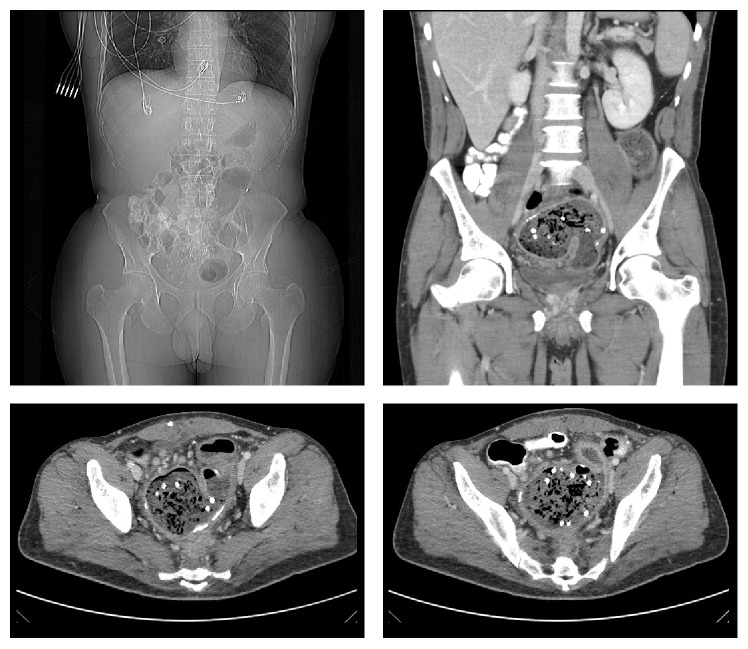
CT scan showing thickening and stranding of the J-pouch and rectum, with obliteration of the rectal fat planes, along with mesenteric lymphadenopathy. There was proximal dilatation with partial air-fluid levels within the small bowel, concerning for partial obstruction.

**Figure 2:**
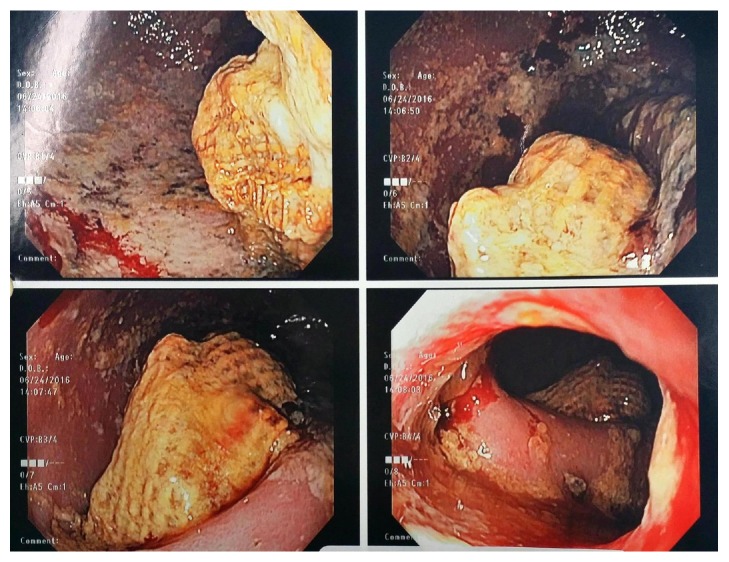
A flexible sigmoidoscopy shows inflamed mucosa with ulceration and adherent mucus. A large foreign object was visualized in the pouch.

**Figure 3:**
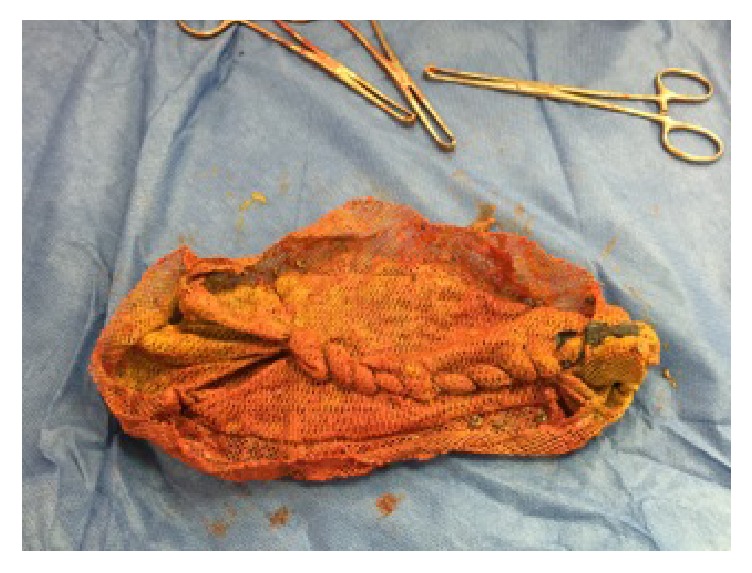
The mesh extracted from the J-pouch retaining tacks and sutures attached from the original placement one year previously.

The patient described his pain as intermittent and vague in nature, starting from the suprapubic area and spreading all over his abdomen, rated 6/10. The pain was not associated with fever, nausea, or vomiting. The patient had been having such episodes since his hernia repair but they increased in intensity over the past three weeks. He also had chronic diarrhea with a small amount of blood when he wipes. Physical exam showed a midline incision scar with bulging when coughing and diffuse tenderness all over the abdomen most prominently over the suprapubic/lower abdominal area. The patient looked ill in mild distress and had positive bowel sounds and tachycardia. Initial lab results showed mild normocytic anemia (HGB: 10.5, WBC: 9800, PLT: 579, HCT: 32, and MCV: 70). CT scan (Figure 1) showed thickening and stranding of the J-pouch and rectum, with obliteration of the rectal fat planes, along with mesenteric lymphadenopathy. There was proximal dilatation with partial air-fluid levels within the small bowel, concerning for partial obstruction. A flexible sigmoidoscopy (Figure 2) showed inflamed mucosa with ulceration and adherent mucus. Multiple biopsies were obtained. A large foreign object was visualized in the pouch. It had a rope-like lattice appearance on its outside; the inner contents were not well visualized. The foreign object was not removed then due to the uncertainty of its origin and extent. The patient was then taken to the operating room for pouch exam under anesthesia where the foreign body was removed in its entirety (Figure 3). The object turned out to be a mesh with tacks and sutures from the original placement one year before. The patient's pain resolved immediately after the procedure and his bowel function returned to normal. The remainder of his postoperative course was unremarkable. He was discharged 2 days after his procedure.

## 3. Discussion

Review of the literature for mesh migration/erosion case reports showed 86 cases in 79 reports published between 1990 and 2015. There were 23 reported cases of erosions, 24 cases of fistulas, 38 cases of migrations, and one case of coincidental fistula and mesh migration. Destinations for migration/erosion included bowel (30), bladder (14), peritoneum/peritoneal cavity (8), stomach (7), esophagus (3), right lower bronchus (1), scrotum (2), and adnexa (1). Fistulas reported were found between bowel and skin (15), bowel and bladder (2), bowel and another segment of bowel (1), bowel and scrotum (1), and bladder and skin (2). The median time calculated between hernia repair and presentation was 48 months (range: 2–360 months).

Mesh migration is defined as the whole mesh displacing into the organ. Mesh erosion is defined as partial mesh perforation into the organ while a portion is still outside. Mesh fistulation is defined as erosion into two organs causing a tract formation between them or abscess formation that erodes to another organ.

Mesh complication presentations vary drastically from incidental findings to mimicking cancer. Still, common themes are shared, including abdominal pain, gastrointestinal bleeding, bowel obstruction, and diarrhea.

The most common complication for all reported hiatal hernias (*n* = 10) was displacement to the stomach (70%, 7/10) with symptoms of dysphagia. Inguinal hernia mesh repairs (*n* = 41) are most frequently displaced to the bladder (27%, 11/41) while presenting with urinary tract infections or hematuria. Ventral/incisional hernias (*n* = 24) routinely displaced to the bowel (92%, 22/24), presenting with bowel obstruction or abdominal pain. The median time to presentation was 24, 48, and 60 months for hiatal, inguinal, and ventral/incisional hernias, respectively. As such, despite erosion, fistula, and migration being rare complications, healthcare providers must remain vigilant of the unpredictability of mesh displacement and other serious complications after hernia repair with seemingly unrelated symptoms.

Table  1 in Supplementary Material available online at https://doi.org/10.1155/2017/3617476 reviews the 86 cases of mesh displacement reported between 1990 and 2015. Mesh and procedure types, the various destinations, the main symptoms, and time of presentations are made accessible for future investigators.

The patient in this case presented to the emergency department with nonspecific symptoms following an ileoanal anastomosis in 2012 and an incisional ventral hernia repair with mesh in 2015. The time to presentation for mesh migration (<12 months) was shorter than the median in our review of the literature (48 months). Interestingly, the mesh migration occurred within 1 year of operation, which is a common time frame for pouchitis to develop after ileoanal anastomosis construction (15–18% within 1 postoperative year) [11, 12]. It is worth noting that both pouchitis and mesh migration are associated with postoperative inflammation.

Despite unclear pathophysiology, mesh migration is presumed to be due to inflammation caused by the foreign body, combined with rejection or displacing forces leading to slow erosion of tissue from the prosthesis [5, 13, 14]. It is unclear whether the history of an abdominal surgery with high incidence of pouchitis is related to the unusual presentation of mesh migration to the J-pouch, but similarities in pathophysiology warrant further investigation into operative procedures, materials, and postoperative prevention that may optimize outcomes for patients with specific surgical morbidities [15, 16]. Improvements remain to be made in the balance between durability, foreign body reaction, amount of material, chronic pain, and time to recovery [17].

## Supplementary Material

Table S1: Features for 86 reported cases of mesh displacement between 1990 and 2015. Mesh and procedure types, destinations, symptoms, and time of presentations are described.
