# Supplementary material for: Mesh Migration into the J-Pouch in a Patient with Post-Ulcerative Colitis Colectomy: A Case Report and Literature Review
Source: Case Rep Surg. 2017 Nov 23;2017:3617476. doi: 10.1155/2017/3617476 (PMC5733182; doi:10.1155/2017/3617476)
Supplement: Supplementary file 1 — Table S1: Features for 86 reported cases of mesh displacement between 1990 and 2015. Mesh and procedure types, destinations, symptoms, and time of presentations are described. [file 3617476.f1.docx]

|  | Publication Writer | Year | Hernia Type | Patient Age | gender | Lap/Open | Procedure Type | Mesh Type | Problem | Destination | Main symptom | Time (mo.) |
| --- | --- | --- | --- | --- | --- | --- | --- | --- | --- | --- | --- | --- |
| 1 | I. Pros | 1990 | Incisional hernia | N/A | M | Open | N/A | Silastic plate synthetic mesh | Fistula | Ileoovesical fistula | N/A | 60 |
| 2 | M. H. Seelig | 1995 | Incisional hernia | N/A | N/A | Open | N/A | Marlex | Fistula | Enterocutaneous fistula | N/A | N/A |
| 3 | K. Miller | 1996 | Inguinal hernia | 67 | M | Lap | TAPP | Polypropylene | Fistula | Ileocutaneous fistula | Suppurative drainage and chills | 12 |
| 4 | ROBERT H. HUME | 1996 | Inguinal hernia | 42 | M | Lap | N/A | Marlex | Erosion | Bladder | Hematuria | 48 |
| 5 | J. Majeski | 1998 | Ventral hernia | N/A | M | Open | N/A | Wire mesh | Migration | Small intestine | Bowel obstruction | 360 |
| 6 | R. A. Dieter | 1999 | N/A | 45 | M | Open | N/A | N/A | Migration | Scrotum | Large tender mass in the scrotum | N/A |
| 7 | John A. Chuback | 2000 | Inguinal hernia | 69 | M | Open | N/A | N/A | Migration | Small intestine | Bowel obstruction | 24 |
| 8 | A. J. Aldridge | 2001 | Paracolostomy hernia | 85 | M | Open | N/A | Polypropylene | Erosion | Colon | Parastomal pain | 26 |
| 9 | E. Deligné | 2001 | Incisional hernia | 68 | M | Open | N/A | Wire mesh | Erosion | Small intestine | Bowel obstruction, 2 years of anemia and melena | 276 |
| 10 | Yukihiko Tokunaga | 2001 | Inguinal hernia | 83 | M | Open | N/A | N/A | Erosion | Peritoneum | Bloody stool | 72 |
| 11 | J. Losanoff | 2002 | Incisional hernia | 52 | M | Open | onlay | Polypropylene | Fistula | Entero-colocutaneous fistula | Fluctuating painful mass in the left anterior abdominal wall | 120 |
| 12 | T. Acar | 2002 | Incisional hernia | 78 | M | Open | preperitoneal | Polypropylene | Fistula | Enterocutaneous fistula | Chills and fever and a suppurative draining | 132 |
| 13 | Roberto Ferrone | 2003 | Inguinal hernia | 67 | M | Open | N/A | Polypropylene | Migration | Peritoneum | Bowel obstruction | 36 |
| 14 | A. A. Riaz | 2003 | Incisional hernia | 57 | F | Open | N/A | N/A | Erosion | Bladder | Frank hematuria | N/A |
| 15 | P. Lauwers | 2003 | Inguinal hernia | 56 | M | Open | N/A | N/A | Erosion | Sigmoid/bladder | Abscess/bladder mass | 24/48 |
| 16 | M. Benedetti | 2004 | Inguinal hernia | 67 | M | Open | N/A | Polypropylene | Fistula | Sigmoid and abdominal wall | Abdominal pain, minimal and sporadic episodes of rectal bleeding | 24 |
| 17 | D. D. Nowak | 2004 | Inguinal hernia | 30 | M | Open | N/A | N/A | Migration | Scrotum | Bowel obstruction | 6 |
| 18 | V. Ott | 2004 | Incisional hernia | 52 | M | Open | N/A | Mersilene | Fistula | Enterocolocutaneus fistula | Malodorous drainage | 96 |
| 19 | Matthew L. Moorman | 2004 | Inguinal hernia | 50 | M | Open | overlay | PerFix plug | Migration | Preperitoneal space | Vague left lower quadrant pain | 18 |
| 20 | Jørgen Bjerggaard Jensen | 2004 | Inguinal hernia | 77 | M | Lap | N/A | Polypropylene | Erosion | Bladder | Recurrent UTI | 84 |
| 21 | Alper Celİk | 2005 | Inguinal hernia | 48 | M | Lap | TAPP | Polypropylene | Migration | Colon | Persistent watery diarrhea | 6 |
| 22 | A. Agrawal | 2005 | Inguinal hernia | 71 | M | Lap | TAPP | Polypropylene | Migration | Bladder | Recurrent UTI | 72 |
| 23 | Lidia Libretti | 2006 | Congenital diaphragmatic hernia | 8 | F | Open | N/A | N/A | Migration | Right lower bronchus | Recurrent pulmonary infections and hemoptysis. | 84 |
| 24 | Pradeep K. Chowbey | 2006 | Inguinal hernia | 45 | M | Lap | TEP | N/A | Fistula | Vesicocutaneous fistula | Persistant drainage | N/A |
| 25 | Peter Ojo | 2006 | Inguinal hernia | 63 | M | Open | N/A | N/A | Migration | Cecum | Suspected colonic malignancy (weight loss, anorexia, fatigue, and a palpable right lower quadrant mass) | 96 |
| 26 | J. W. Murphy | 2006 | Inguinal hernia | 59 | M | Open | N/A | PerFix plug | Fistula | Sigmoid | Abdominal pain | 24 |
| 27 | Tru Ngo | 2006 | Inguinal hernia | 64 | M | Lap | N/A | Polypropylene | Erosion | Bladder | Recurrent UTI | 156 |
| 28 | Pedro Hergueta-Delgado | 2006 | Hiatal hernia | 74 | F | Lap | nissen fundoplication | PTFE | Migration | Stomach | Dysphagia | N/A |
| 29 | Rup Goswami | 2007 | Inguinal hernia | 66 | M | Lap | TAPP | N/A | Erosion | Cecum | Intermittent diarrhea | 84 |
| 30 | D. Borchert | 2007 | Obturator hernia | 87 | F | Open | N/A | Polypropylene | Migration | Small intestine | Bowel obstruction | 84 |
| 31 | Osman Kurukahvecioglu | 2007 | Inguinal hernia | 50 | M | Lap | onlay | PTFE | Migration | Bladder | UTI and frank hematuria | 48 |
| 32 | Sanjeev Dutta | 2007 | Hiatal hernia | 12 | M | Lap | N/A | PTFE | Erosion | Esophagus | Obstruction | 108 |
| 33 | Antonio Di Muria | 2007 | Umbilical hernia | 61 | M | Open | N/A | N/A | Migration | Small intestine | Bowel obstruction | 72 |
| 34 | Christopher L. Stout | 2007 | Inguinal hernia | 76 | M | Open | N/A | N/A | Erosion | Small intestine | Volvulus | 24 |
| 35 | D. J. Lo | 2008 | Inguinal hernia | 56 | M | Open | N/A | Prolene hernia system (PHS) | Erosion | Peritoneal cavity | Bowel obstruction and pain | 18 |
| 36 | M. A. Rettenmaier | 2008 | Inguinal hernia | 79 | F | Open | N/A | Polypropylene | Migration | Adnexa | Suspected adnexal malignancy | 36 |
| 37 | Gavin A. Falk | 2009 | Ventral hernia | 64 | F | N/A | N/A | Polypropylene | Migration | Bowel? | Bowel obstruction | N/A |
| 38 | Adel Y. Daas | 2009 | Inguinal hernia | N/A | N/A | N/A | N/A | Polypropylene | Migration | Cecum | N/A | N/A |
| 39 | S. G. Barreto | 2009 | Inguinal hernia | 54 | M | Open | preperitoneal | Kugel mesh | Fistula | Colovesical Fistula (sigmoid) | Septicemia, pneumaturia and left iliac fossa pain. | 48 |
| 40 | Eric J. Hazebroek | 2009 | Hiatal hernia | 80 | F | Open | Nissen fundoplication | Composite PTFE/ePTFE | Erosion | Stomach | Dysphagia and regurgitation | 4 |
| 41 | A. Hamouda | 2009 | Inguinal hernia | 67 | M | Lap | TEP | prolene | Erosion | Bladder | Recurrent sepsis, groin swelling, and lower urinary tract symptoms | 144 |
| 42 | M. Foda | 2009 | Incisional hernia | 50 | M | Open | N/A | ePTFE | Fistula | Enterocutaneous fistula | Fever and abdominal pain | 36 |
| 43 | E. C. Nelson | 2010 | Ventral hernia | 65 | M | Open | N/A | Composite | Fistula | Ileocutaneous fistula | Yellow drainage from fistula | 60 |
| 44 | A. Moussi | 2010 | Incisional hernia | 66 | M | Open | N/A | Polyester | Fistula | Enterocutaneous fistula | Gas gangrene of the abdominal wall | 72 |
| 45 | E. Steinhagen | 2010 | Incisional hernia | 67 | M | Open | N/A | GORE-TEX | Migration | Small intestine | Bowel obstruction | 12 |
| 46 | Ming-Jenn Chen | 2010 | Inguinal hernia | 79 | M | Open | N/A | Polypropylene | Erosion | Small intestine | Lower abdominal pain | 24 |
| 47 | B. Szitkar | 2010 | Inguinal hernia | 54 | M | Lap | N/A | N/A | Migration | Colon | abdominal pain | 7 |
|  |  | 2010 | Femoral hernia | 56 | F | Lap | N/A | Mersilene | Erosion | Bladder | Bowel obstruction | 144 |
|  |  | 2010 | Incisional hernia | 76 | F | Open | N/A | Mersilene | Fistula | Jejunum and the abdominal wall | Abscess | 324 |
| 48 | M. Carpelan-Holmström | 2010 | Hiatal hernia | 53 | M | Lap | nissen fundoplication | PTFE/ePTFE | Migration | Stomach | Spontaneous evacuation - dysphagia and weight loss | 24 |
| 49 | M. Chan  d | 2010 | Incisional hernia | 67 | F | Lap | N/A | ParietexTM Composite (PCO) Mesh | Erosion | Bladder | UTI and frank hematuria | several years |
| 50 | J. P. T. Novaretti | 2010 | Inguinal hernia | 68 | M | Open | N/A | Polypropylene | Migration | Bladder | Recurrent UTI | 240 |
| 51 | M. Horzic | 2010 | Incisional hernia | 58 | M | Open | N/A | Polypropylene/ePTFE | Migration/fistula | Small intestine and colon fistula | Spontaneous evacuation | 24 |
| 52 | I. Yilmaz | 2011 | Inguinal hernia | 36 | M | Open | N/A |  | Migration | Sigmoid mesentry | Bowel obstruction | 36 |
| 53 | Divyangkumar Gandhi | 2011 | Incisional hernia | 56 | F | Open | inlay | Polypropylene | Erosion | Cecum | Chronic abdominal pain | 168 |
| 54 | Q. Arroyo | 2011 | Hiatal hernia | 71 | F | Lap | N/A | ePTFE | Migration | Esophagus | Progressive dysphagia and weight loss | 24 |
| 55 | Marc A. Bjurlin | 2011 | Femoral hernia | 76 | F | Open | N/A | N/A | Fistula | Vesicocutaneous fistula | Inguinal drainage. UTIs, urinary symptoms | 36 |
| 56 | Kevin A. Karls | 2011 | Inguinal hernia | 75 | M | Lap | N/A | Polypropylene | Erosion | Sigmoid | Lower GI bleeding | 144 |
| 57 | Ashish Kumar Jha | 2012 | Lumbar hernia | N/A | M | N/A | N/A | N/A | Fistula | Colocutaneous | N/A | N/A |
| 58 | V. Porziella | 2012 | Hiatal hernia | 47 | F | Open | Nissen fundoplication with PTFE dual-mesh cruroplasty | PTFE | Migration | Stomach | Subacute onset of severe dysphagia and weight loss | N/A |
| 59 | Véronique De Moor | 2012 | Hiatal hernia | N/A | N/A | Open | N/A | N/A | Erosion | Esophagus | Epigastric pain, dysphagia | N/A |
|  |  | 2012 | Hiatal hernia | N/A | N/A | Open | N/A | N/A | migration | Stomach | Epigastric pain, dysphagia | N/A |
|  |  | 2012 | Hiatal hernia | N/A | N/A | Open | N/A | N/A | Erosion | Stomach | Epigastric pain, dysphagia | N/A |
| 60 | Marinko Zuvela | 2012 | Inguinal hernia | 42 | M | Open | N/A | Prolene hernia system (PHS) | Fistula | Appendix | Recurrent cellulitis and an abscess in the right groin | 72 |
|  |  | 2012 | Inguinal hernia | 76 | M | Open | N/A | Prolene hernia system (PHS) | Fistula | Appendix | Painful swelling in the right groin, pus drainage | 84 |
|  |  | 2012 | Inguinal hernia | 60 | M | Open | N/A | Prolene hernia system (PHS) | Fistula | Sigmoid | Pus drainage | 96 |
| 61 | G. Voisard | 2013 | Incisional hernia | 73 | M | Open | N/A | Composite polypropylene/ePTFE | Migration | Small intestine | Microcytic anemia and abdominal pain | 60 |
| 62 | Sk Tiwari | 2013 | Inguinal hernia | 70 | M | Open | onlay | Polypropylene | Migration | Peritoneum | Bowel obstruction | 240 |
| 63 | S. G. Millas | 2013 | Supraumbilical hernia | 41 | F | Open | N/A | Ventralex composite mesh | Migration | Sigmoid colon | Chronic abdominal pain | 24 |
| 64 | S. Olmi | 2013 | Incisional hernia | 59 | M | Lap | N/A | PTFE | Migration | Small intestine | Bowel obstruction | 18 |
| 65 | A. Garioud | 2013 | Periumbiical hernia | 59 | M | Open | N/A | N/A | Erosion | Cecum-Incomplete migration of surgical mesh embedded in the colonic mucosa was diagnosed | Incidental finding | 30 |
| 66 | S. Olmi | 2013 | Incisional hernia | 59 | M | Open | N/A | PTFE | Migration | Small intestine | Bowel obstruction | 18 |
| 67 | Ian Fukudome et al | 2014 | N/A | 58 | F | N/A | N/A | N/A | Migration | Transverse Colon | N/A | N/A |
| 68 | S. Yamamoto | 2014 | Inguinal hernia | 72 | M | Open | N/A | Polypropylene | Migration | Peritoneal cavity | Bowel obstruction | 24 |
| 69 | W. B. Li | 2014 | Inguinal hernia | 49 | M | Lap | N/A | N/A | Migration | Bladder | Recurrent pain and frank hematuria | 36 |
|  |  | 2014 | Inguinal hernia | 47 | M | Lap | N/A | N/A | Migration | Bladder | Recurrent frank hematuria | 48 |
| 70 | M. Bodenbach | 2014 | Inguinal hernia | 48 | F | Lap | TAPP | Polypropylene | Migration | Bladder | Recurrent UTI | 36 |
| 71 | Martin Rasmussen | 2014 | Ventral hernia | 63 | F | Lap | N/A | N/A | Migration | Colon | N/A | N/A |
| 72 | Yann-Rong Su | 2014 | Ventral hernia | 77 | M | Open | N/A | N/A | Fistula | Enterovesical fistula | Lower urinary tract symptoms and microhematuria | 60 |
| 73 | D. Acin-Gandara | 2014 | Hiatal hernia | 68 | F | Lap | nissen fundoplication | Composite PTFE/ePTFE | Migration | Stomach | Dysphagia | 6 |
| 74 | Wiadomości lekarskie | 2015 | Bilateral inguinal hernia | N/A | N/A | Lap | TAPP | N/A | Fistula | Sigmoid-cutaneous | N/A | 60 |
| 75 | Luke S. Scheuer | 2015 | Inguinal hernia | 65 | M | Open | N/A | N/A | Migration | Peritoneal cavity | Incidental finding | 240 |
| 76 | Arshad Mehmood Malik | 2015 | Incisional hernia | 50 | M | Open | N/A | N/A | Fistula | Enterocutaneous fistula | Fecal discharge through the fistula | 60 |
| 77 | Yoshiaki Mizuguchi | 2015 | Inguinal hernia | 57 | M | Open | N/A | N/A | Fistula | Entero-subcutaneous fistula (cecum) | Swelling and pain in his right groin | 156 |
| 78 | Tong-min Xue | 2015 | Inguinal hernia |  |  | Lap | N/A | N/A | Erosion | Small intestine | N/A | N/A |
| 79 | Jad A. Degheili | 2015 | Inguinal hernia | 75 | M | Lap | TAPP | Polypropylene | Fistula | Sigmoid to scrotal fistula | Sepsis and septic shock | 2 |
